# Efficacy of copper-impregnated hospital linen in reducing healthcare-associated infections: A systematic review and meta-analysis

**DOI:** 10.1371/journal.pone.0236184

**Published:** 2020-07-20

**Authors:** Tingting Fan, Li Shao, Xingzhen Wang, Ping Ren

**Affiliations:** Zaozhuang Hospital of Traditional Chinese Medicine, Zaozhuang, Shandong, P.R. China; Rabin Medical Center, Beilinson Hospital, ISRAEL

## Abstract

**Background:**

Healthcare-associated infections (HAI) are a significant burden on the healthcare system. Recent research has suggested the role of copper in reducing HAI. The purpose of this study was to systematically search literature and pool data from studies evaluating the efficacy of copper-impregnated hospital linen in reducing HAI.

**Methods:**

We carried out a systematic electronic search of PubMed, ScienceDirect, BioMed Central, Springer, Embase, and Google Scholar databases for controlled studies evaluating the efficacy of copper-impregnated linen in reducing the incidence of HAI. The last search was carried out on 15^th^ February 2020.

**Results:**

Six studies were included. There was no restriction on the type of organism causing HAI in three studies while three trials reported HAI from *Clostridioides difficile* and multi-drug resistant organisms (MDRO). A meta-analysis of six studies indicated the use of copper-impregnated linen did not reduce the risk of HAI [Incidence rate ratio (IRR):0.66, 95% CI:0.28–1.58, p = 0.36, I^2^ = 100%)]. On subgroup analysis, while pooled data from three studies HAI indicated a statistical significant reduction in all-HAI with copper-impregnated linen (IRR:0.76, 95% CI:0.75–0.77, p<0.00001, I^2^ = 0%), no such difference was seen when HAI was defined as infection by *Clostridioides difficile* and MDROs only (IRR:0.57, 95% CI:0.12–2.75, p = 0.48, I^2^ = 99%). Meta-regression analysis for study duration and number of days of hospitalization did not demonstrate any influence on the overall effect size. On sensitivity analysis, there was no change in the significance of results after the sequential exclusion of every study.

**Conclusion:**

Current evidence on the use of copper-impregnated linen to reduce HAI is conflicting. Our results indicate that copper-impregnated linen may reduce HAI, but there is still no evidence of such an effect regarding infections caused by MDRO or *Clostridioides difficile*. The overall quality of evidence is not high. Homogenous high-quality studies are required to strengthen the evidence on this subject.

## Introduction

Healthcare-associated infections (HAI) are a significant burden on the healthcare system. An estimated 648,000 to 1.7 million hospitalized patients in the USA are affected by HAI [1]. A recent systematic review suggests the prevalence of HAI be 3.12% in mainland China, with rates as high as 26.07% in adult intensive care units (ICU) [2]. HAI not only increases hospitalization costs but has a significant effect on the patient’s morbidity and mortality [3]. The mortality rates are significantly increased if they are caused by multi-drug resistant organisms (MDRO) [4].

Contaminated hospital surfaces are an important source of pathogen transmission. Objects which are in greater vicinity to patients are more likely to be contaminated with infectious pathogens [5]. Hospital linen is in close contact with patients and may be highly contaminated with substances like blood, skin, stool, urine, vomitus, and other body tissues and fluids [6]. According to Ohl et al [7], about 92% of hospital privacy curtains are contaminated with potentially pathogenic bacteria, such as Methicillin-resistant *Staphylococcus aureus* (MRSA) and Vancomycin-resistant *Enterococci* (VRE) within one week of use. Such pathogens on hospital linen may persist and in the presence of a favorable microenvironment can contribute to HAI [8]. Recent research in microbiology and infection control has focused on the role of copper in reducing HAI [9]. Several studies have been conducted evaluating the role of copper-impregnated surfaces as well as copper-impregnated hospital clothing in reducing the incidence of HAI [10–13]. A systematic review and meta-analysis published in 2017 have suggested that the use of antimicrobial copper alloys in replacement of high-touch surfaces may reduce the incidence rate of HAI [14]. However, to the best of our knowledge, to date, no systematic review has examined the effect of copper-impregnated textiles on such infections. Therefore, the purpose of this study was to systematically search literature and pool data from studies evaluating the efficacy of copper-impregnated hospital linen in reducing HAI.

## Material and methods

### Search strategy

We carried out a systematic electronic search of PubMed, ScienceDirect, BioMed Central, Springer, Embase and Google Scholar databases. The search was carried out from 1^st^ January 1990 to 15^th^ February 2020. Our search strategy included the following search terms: “copper”; “clothing”; “linen”; “bedsheets”; “dress”; “antimicrobial”; “infection” and “hospital”. The search strategy and results of the PubMed database are presented in S1 File. Language restriction was not placed for identifying studies. Furthermore, we performed a manual search of bibliography of included studies to look for any missed studies. The literature search was performed by two separate reviewers. They evaluated the studies initially at the title and abstract level. Studies fulfilling inclusion criteria or potentially fulfilling inclusion criteria were analyzed by their full texts for a final decision. Any conflicts between the two reviewers were resolved by discussion. Guidelines of the PRISMA statement (Preferred Reporting Items for Systematic Reviews and Meta-analyses) [15] and Cochrane Handbook for Systematic Reviews of Intervention [16] were followed during the conduct of this review, except for protocol registration.

### Selection criteria

Inclusion criteria were framed based on the PICOS (Population, Intervention, Comparison, Outcome, and Study design) framework. We included the following types of studies: randomized controlled trials (RCTs), cluster RCTs, before-after studies, and case-control studies. The study *population* was to include any group of hospitalized patients in acute care or long term care. No restriction was placed for the reason for hospitalization. *The intervention* was the use of copper-impregnated hospital linen which could include bedsheets, pillow covers, towels, patient clothing, blankets, or any other textile used for hospitalized patients. Studies were to *compare* the intervention with similar linen used for a similar cohort of patients but not impregnated with copper. *Outcomes* were to include the incidence of HAI. The definition of HAI was as per the included study. Other surface cleaning or infection control measures were to be unchanged for both the study cohorts. We excluded studies evaluating any other antimicrobial agent impregnated linen. Studies without a control group and studies not reporting required data were also excluded. In the case of publications with duplicate data, the study published earlier was included.

### Data extraction and outcomes

Data were extracted from the included studies by two reviewers. The following details were obtained using a pre-prepared data collection form: Authors, publication year, study type, location of the study, specific ward data, duration of study, type of linen used, other infection control measures, laundry protocol, number of patients studied, total hospitalization days, age group of patients, outcomes, and definition of outcomes. The primary outcome was the incidence of HAI with the use of copper-impregnated linen vs regular linen. The secondary outcome measure was organism-specific HAI [due to MDRO and *Clostridioides difficile* (formerly known as *Clostridium difficile*)]. Any other outcomes reported by the included studies were analyzed descriptively.

### Risk of bias

The risk of a bias assessment tool for non-randomized studies (RoBANS) was used [17] for assessing quality of included studies. Studies were assessed for: selection of participants, confounding variables, intervention measurements, blinding of outcome assessment, incomplete outcome data, and selective outcome reporting.

### Statistical analysis

HAI data were presented as incidence rates in the included studies. We calculated the incidence rate ratios (IRR) with 95% confidence intervals (CI) for studies included in the meta-analysis. Study estimates were then combined using inverse variance-weighted averages of logarithmic IRRs in a random-effects model. Review Manager (RevMan, version 5.3; Nordic Cochrane Centre [Cochrane Collaboration], Copenhagen, Denmark; 2014) was used for the meta-analysis. Anticipating methodological heterogeneity in the included trials, we used a random-effects model to calculate the pooled effect size. Heterogeneity was calculated using the I^2^ statistic. I^2^ values of 25–50% represented low, values of 50–75% medium and >75% represented substantial heterogeneity. A sensitivity analysis was carried out to evaluate the influence of each study on the result of the primary outcome. Meta-analysis was conducted only if at least 3 studies reported similar data. We also performed a random-model meta-regression analysis assessing the influence of the total number of hospitalization days and the duration of the study on the log-transformed values of IRR. Meta-essentials was used for performing the meta-regression analysis [18]. Funnel plots were not used to assess publication bias due to limited number of studies included in the meta-analysis (<10).

## Results

**Fig 1:**
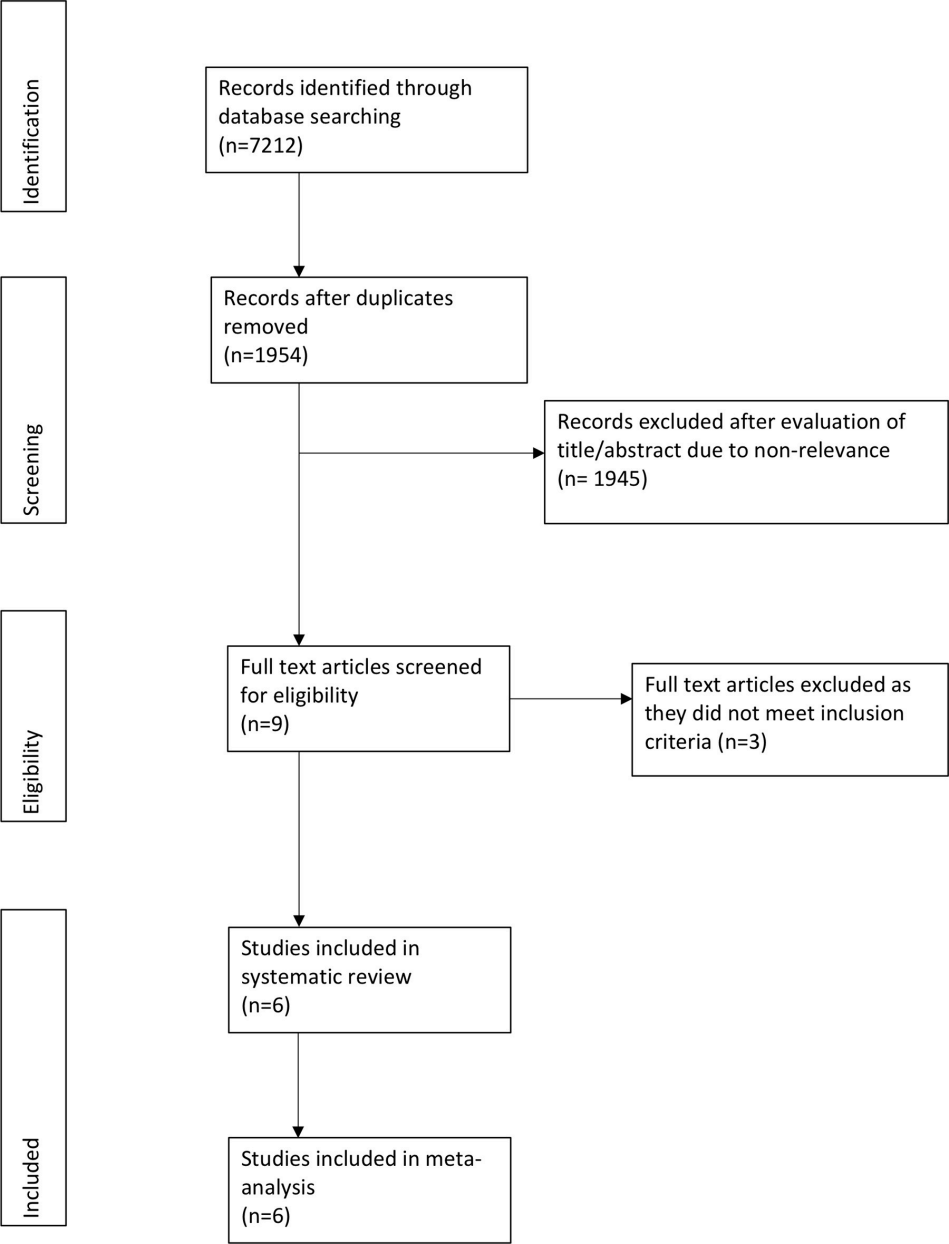
Study flow chart.

The number of search results after the database search and study flow is presented in Fig 1. nine studies were selected for full-text analysis. Three studies were excluded as 1 study did not use copper-impregnated linen [19], one study did not report relevant outcomes [20] and one trial reported duplicate data [21]. A total of six studies were finally included in this review and meta-analysis [13,22–26].

Details of included trials are presented in Table 1. Four studies were before-after studies comparing outcomes of copper-impregnated linen with regular linen at different periods. Two studies were cross-over RCTs [22,26]. None of the studies were conducted during an outbreak of MDRO HAI or endemic setting. The setup was however similar for both study and control groups. While four studies analyzed data from single hospital setups, Butler [24] evaluated data from six health-care centers. The study and control groups were from the same hospitals setups at all six health-care centers. The duration of the data collection varied from six months to 37 months in the included studies. Linen impregnated with copper were all patients used clothing. In the trial of Sifri et al [25], hard surfaces were also impregnated with copper in addition to hospital linen. Cleaning and infection control measures were reported to be similar for both cohorts in all included studies. The number of hospitalization days was least in the study of Marik et al [22] and highest in the study of Butler [24], as data of six centers were combined in their study. three studies [22–25] utilized the National Healthcare Safety Network (NHSN) definition [24] of HAI, while one study defined HAI according to Embry and Chinnes criteria [27] as well as revised McGeer criteria [28]. In the study of Marcus et al [26], HAI was not clearly defined.

**Table 1:** Characteristics of included studies.

Author, Year	Type of study	Country and Location of study	Area of study	Duration of study in months		Type of linen impregnated with copper	Other infection control measures	Laundry protocol	Number of patients		Total hospitalization days		Mean age		Outcomes assessed
				Study	Control				Study	Control	Study	Control	Study	Control	
Lazary et al [13], 2014	Before-after study	Israel, Tertiary care hospital	Head injury ward	6	6	Bed sheets, pillowcases, patient shirts, patient pants, patient gowns, towels, underpads, and personnel robes	Standard precautions, isolation of patients infected with MDRO, hand hygiene	Same for copper and non-copper linen	51	57	3940	4337	49.7	57	All HAI
Sifri et al [25], 2016	Before-after study	USA, Tertiary care hospital	Acute care ward	10	12	Patient gowns, pillowcases, fitted and flat sheets, washcloths, bath towels, bath blankets, and thermal blankets*	NS	Same for copper and non-copper linen	4704	5257	14479	19177	58.5	60.5	MDRO and *Clostridioides difficile* HAI
Marcus et al [26], 2017	Crossover RCT	Israel, Tertiary care hospital	Ventilator dependent ward	6	6	Hospital linen, patients clothes and towels	Details not specified	Same for copper and non-copper linen	58	54	4159	4050	71.3	69.8	All HAI
Butler [24], 2018	Before-after study	USA, Six healthcare centers	NS	9	9	Patient gowns, pillow- cases, fitted and flat sheets, washcloths, bath towels, bath blankets, and thermal blankets	Isolation precaution, room cleaning and disinfection with validation education and cleaning checklists	NS	NS	NS	94125	81448	NS	NS	MDRO and *Clostridioides difficile* HAI
Madden et al [23], 2018	Before-after study	USA, Acute care hospital	Acute care hospital	27	37	Bed sheets, fitted sheets, pillowcases, towels, and washcloths	NS	NS	NS	NS	25243	29342	NS	NS	MDRO and *Clostridioides difficile* HAI
Marik et al [22], 2020	Crossover RCT	USA, Tertiary care hospital	ICU	10	10	Bed sheets, fitted sheets, pillowcases, underpads, wash cloths, towels, and patient gowns	NS	Same for copper and non-copper linen	637	645	2185	2141	60	60	All HAI and *Clostridioides difficile* HAI

RCT, Randomized controlled trial; MDRO, Multi-drug resistant organisms; NS, not specified; ICU, intensive care unit; HAI, healthcare-associated infections

* included copper-impregnated hospital surfaces as well

### Primary outcome

**Fig 2:**
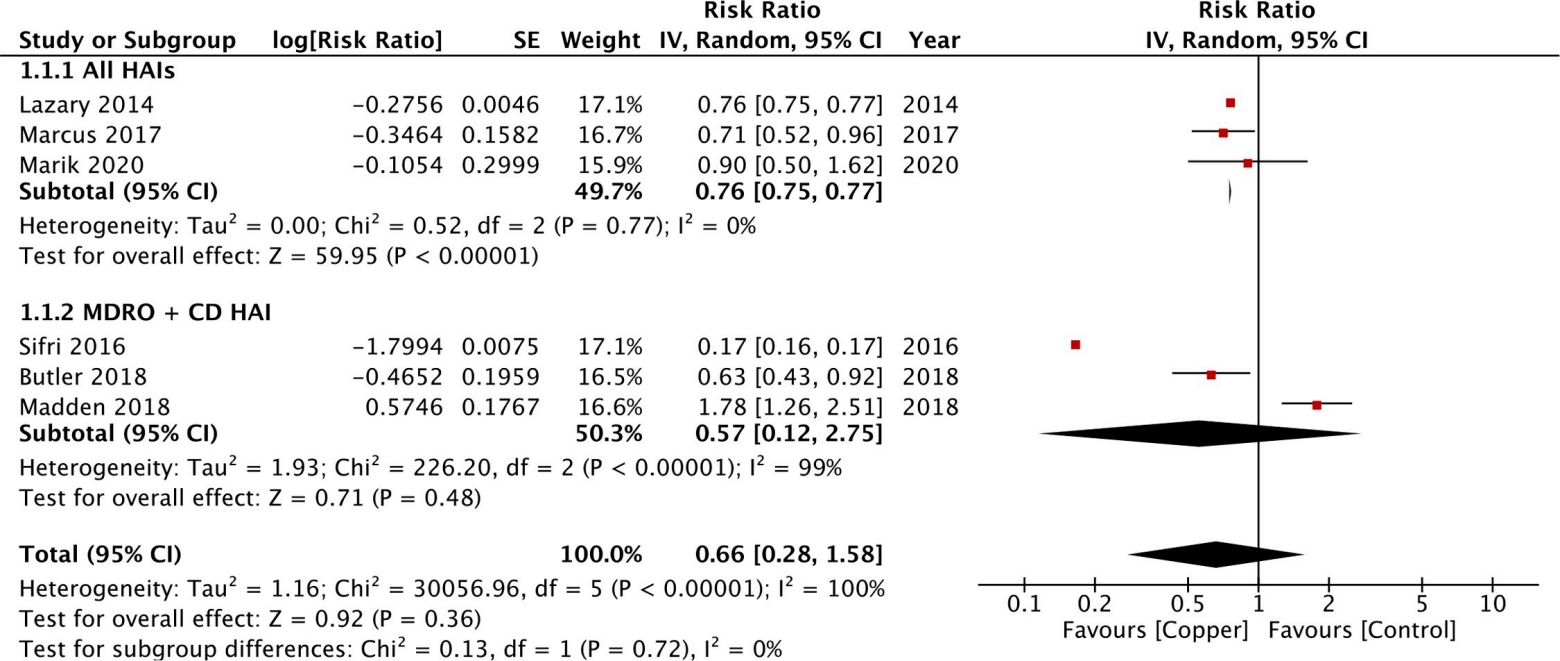
Forest plot of HAI for copper-impregnated linen vs regular linen with sub-group analysis based on definition of HAI.

There was no restriction on the type of organism causing HAI in three studies [13,22,26], while three trials reported HAI from *Clostridioides difficile* and MDROs [23–25]. MDRO definition included MRSA, VRE, extended-spectrum B-lactamase, multidrug-resistant *Acinetobacter baumannii*, and carbapenem-resistant *Enterobacteriaceae* in all three studies. We combined data on these studies in different sub-groups for our analysis. Overall, our meta-analysis of six studies indicated the use of copper-impregnated linen did not reduce the risk of HAI (IRR:0.66, 95% CI:0.28–1.58, p = 0.36, I^2^ = 100%) (Fig 2). On subgroup analysis, while pooled data from three studies indicated a statistical significant reduction in all-HAI with copper-impregnated linen (IRR:0.76, 95% CI:0.75–0.77, p<0.00001, I^2^ = 0%), no such difference was seen when HAI was defined as infection by *Clostridioides difficile* and MDROs (IRR:0.57, 95% CI:0.12–2.75, p = 0.48, I^2^ = 99%) (Fig 2). On sensitivity analysis, there was no change in the significance of results after the sequential exclusion of every study.

### Meta-regression analysis

**Fig 3:**
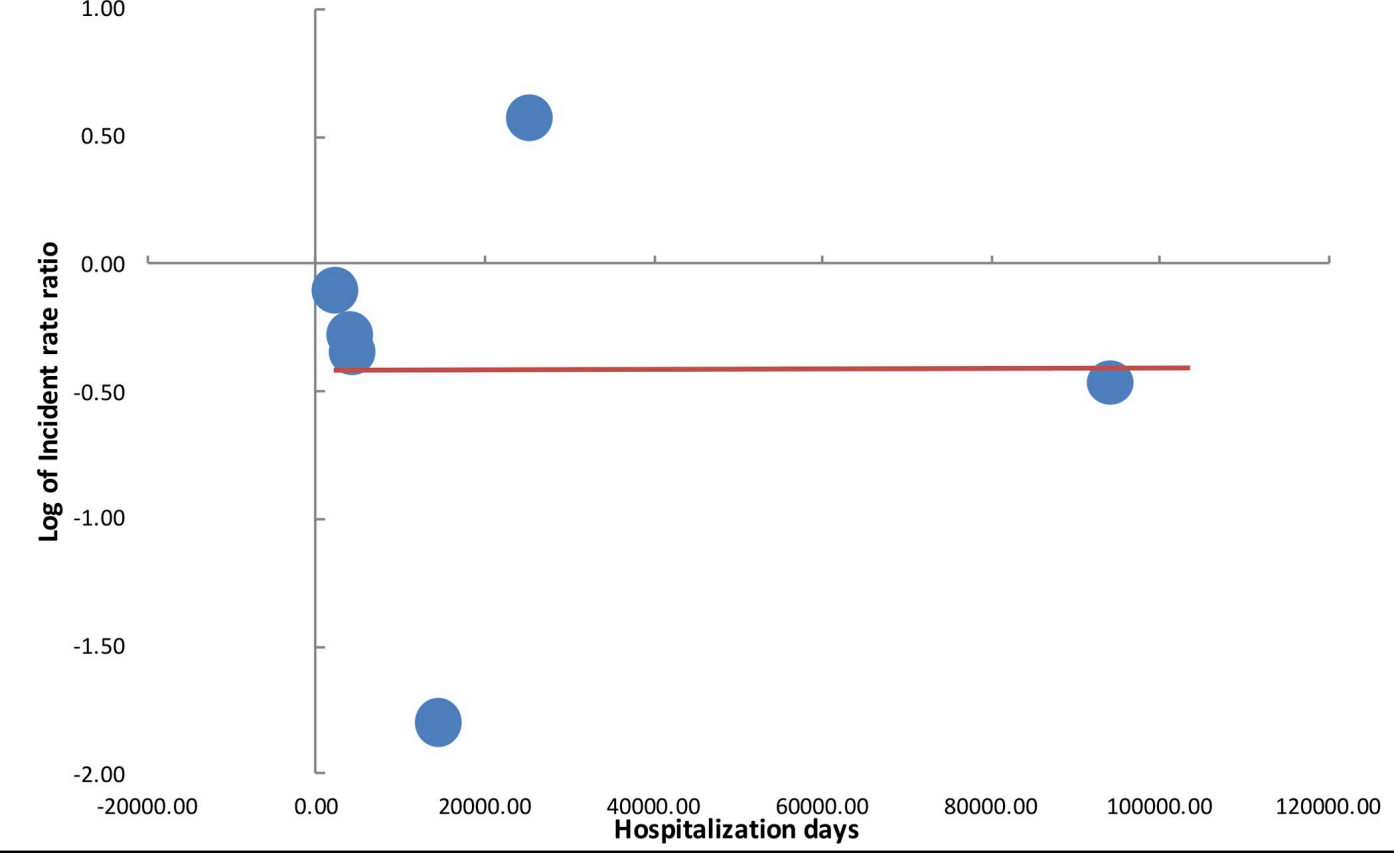
Meta-regression bubble plot for influence of hospitalization days on log of incidence rate ratio of HAI.

**Fig 4:**
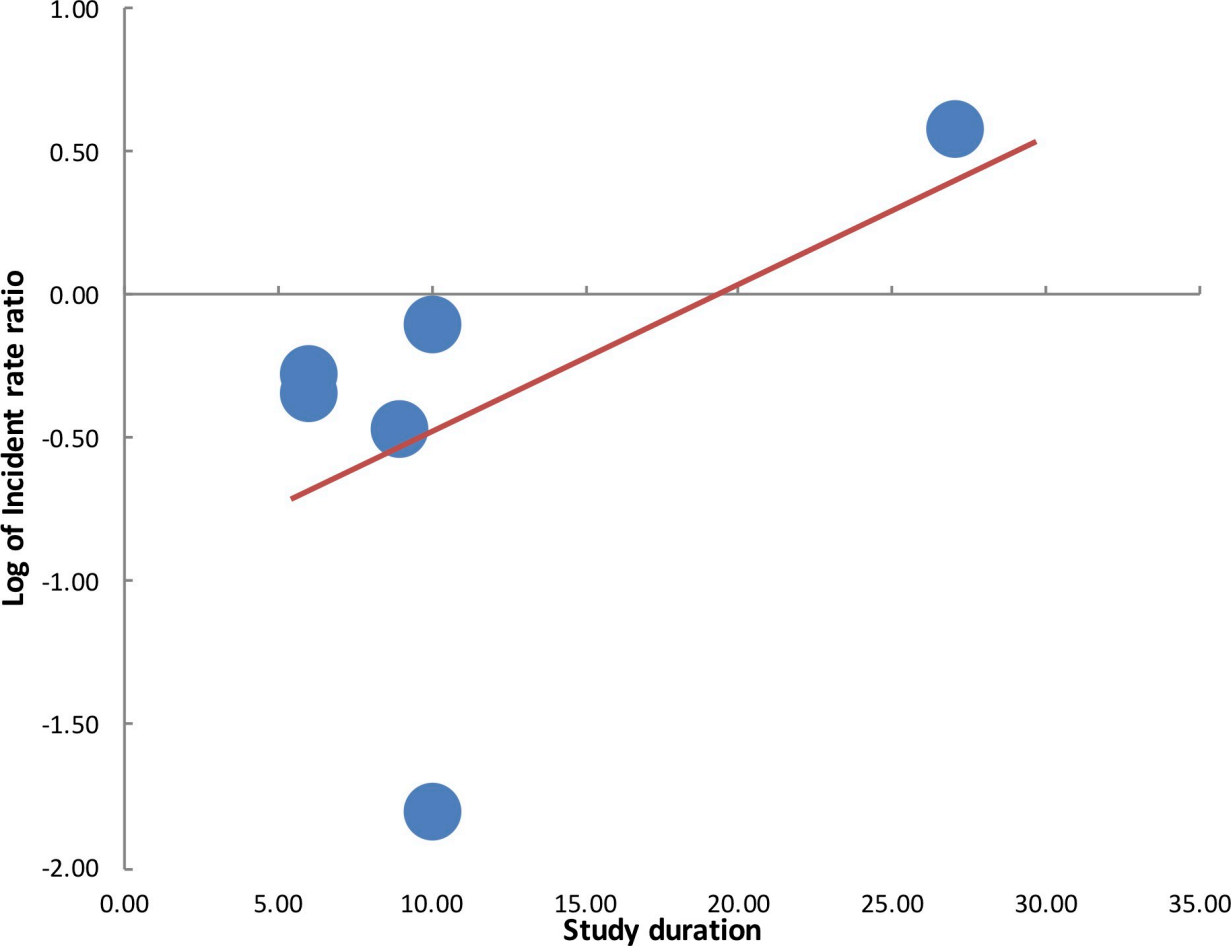
Meta-regression bubble plot for influence of study duration on log of incidence rate ratio of HAI.

Meta-regression analysis for total number of hospitalization days in the study group did not demonstrate any statistical significant influence on the log of IRR (β = 0.000000086; 95% CI: - 0.0000371, 0.0000373; p = 0.99) (Fig 3). Similarly, there was no statistical significant influence on the duration of the study on the effect size (β = 0.05; 95% CI: -0.13, 0.23; p = 0.473) (Fig 4).

### Secondary outcomes and quality of studies

**Fig 5:**
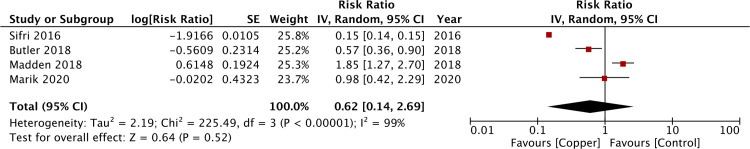
Forest plot of HAI of *Clostridioides difficile* infection for copper-impregnated linen vs regular linen.

**Fig 6:**
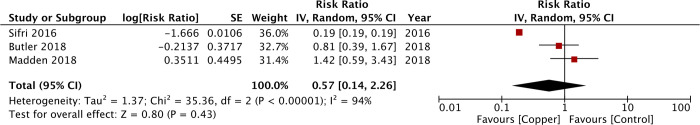
Forest plot of HAI of multi-drug resistant organism (MDRO) infection for copper-impregnated linen vs regular linen.

Four studies reported data on the incidence of HAI by *Clostridioides difficile* and MDROs separately [23–25]. These were pooled together as secondary outcomes. Our analysis did not find any significant reduction of *Clostridioides difficile* infection with the use of copper-impregnated linen (IRR:0.62, 95% CI:0.14–2.69, p = 0.52, I^2^ = 99%) (Fig 5). Similarly, no statistically significant difference was noted in the risk of MDRO HAI with copper-impregnated and regular linen (IRR:0.57, 95% CI:0.14–2.26, p = 0.43, I^2^ = 94%) (Fig 6). The reviewer’s judgement of risk of bias in the included studies is presented in Table 2.

**Table 2:** Risk of bias in included studies.

Study	Selection of participants	Confounding variables	Intervention measurements	Blinding of outcome assessment	Incomplete outcome data	Selective outcome reporting
Lazary et al [13]	High risk	High risk	Low risk	Low risk	Unclear risk	Unclear risk
Sifri et al [25]	High risk	High risk	Low risk	High risk	Unclear risk	Unclear risk
Marcus et al [26]	Low risk	Low risk	Low risk	Low risk	Unclear risk	Unclear risk
Butler [24]	High risk	High risk	Low risk	High risk	Unclear risk	Unclear risk
Madden et al [23]	High risk	High risk	Low risk	High risk	Unclear risk	Unclear risk
Marik et al [22]	Low risk	Low risk	Low risk	Low risk	Unclear risk	Unclear risk

The incidence rate of fever days and the total number of antibiotic days in the study and control groups normalized per 1000 hospitalization days were reported by two studies. Lazary et al [13] reported a significant reduction of fever days (7.1 vs 13.4, p = 0.0085) and the total number of antibiotic days (257.1 vs 382.7, p<0.0001) with the use of copper-impregnated linen vs regular linen. Similar results were reported by Marcus et al [26] for fever days (20.68 vs 46.42, p<0.0001) and the total number of antibiotic days (131.04 vs 170.12, p<0.0001).

Sifri et al [25] reported the incidence of central-line associated bloodstream infections (CLABSI) and catheter-associated urinary tract infections (CAUTI) in their study. The incidence rates of CLABSI were not significantly different between the two groups (3.82 vs 8.91, p = 0.726). Similarly, no statistical significant difference was found in the incidence rates of CAUTI (0 vs 3.78, p = 569). Similarly, Marik et al [22] also reported no difference in CLABSI and CAUTI in their study.

## Discussion

HAI is a global problem contributing to increased morbidity and mortality. Despite the best efforts, HAI may not be 100% preventable. But it is estimated that HAI in the range of 35–55% may be prevented by using multifaceted infection-control methods, irrespective of the country’s financial health [29]. Results of strict infection-control practices have been noted in developed countries like the USA, wherein the point-prevalence of HAI in 199 hospitals was reported to be significantly less in 2015 as compared to 2011 [30]. Our review aimed to examine if the use of one such infection-control measure i.e. copper-impregnated hospital linen leads to reduced incidence of HAI.

The biocidal effect of copper on various pathogens like bacteria, fungi, and viruses has been demonstrated in several in-vitro studies [31,32]. The exact mechanism of action is, however, not fully understood and it is believed that copper causes cell death by several mechanisms like cell membrane permeabilization, membrane lipid peroxidation, protein alteration, and denaturation of nucleic acids [9]. To reduce the incidence of HAI, copper and copper alloys were initially used as surface materials in hospital settings. Many trials have tried to assess the impact of copper surfaces in and around the patients to reduce microbial burden and incidence of HAI [11,12]. Salgado et al [12] evaluated the efficacy of copper alloy surfaces in an RCT of 614 patients managed in three intensive care units (ICU). They reported significantly reduced MRSA and VRE colonization of ICU rooms fitted with copper surfaces and reduced incidence of HAI in patients treated in these rooms. Similarly, Colin et al [11] have reported reduced bacterial contamination of copper alloy door handles and handrails fitted in five French long-term care facilities as compared to controls. In a prospective pilot study, Zerbib et al [10] reported a reduced incidence of hand-transmitted HAI in nursing homes with copper surfaces as compared to controls. To the best of our knowledge, to date, only one meta-analysis has assessed the role of copper in reducing HAI. Pineda et al [14], in a review of 14 studies, analyzing the efficacy of copper-alloys in high-touch surfaces concluded that copper surfaces significantly reduce the risk of HAI (IRR 0.74, 95% CI 0.56 to 0.97). However, the quality of evidence as reported by the authors was low with the upper limit of the CI close to 1. Also, data were pooled from only three studies in their review.

In contrast, our results from 6 studies indicate that copper-impregnated linen may not be able to reduce the risk of HAI (IRR:0.66, 95% CI:0.28–1.58). It is important to note that, in the sub-group analysis based on the definition of HAI, our results were not coherent. In studies restricting data of HAI to MDRO and *Clostridioides difficile*, we did not find any significant difference in the risk of HAI. On the other hand, when data of studies assessing all-HAI was pooled, irrespective of microbial resistance, our results indicated a statistical significant reduction of HAI with the use of copper-impregnated linen. The difference in HAI definition and absence of coherent data collection amongst different centers has been a barrier in defining the overall HAI prevalence as well as the effectiveness of infection-control measures [33]. On further analysis of only MDROs and *Clostridioides difficile* infections separately, our results could not demonstrate any beneficial effect of copper in hospital linen. In this context it is important to note that in-vitro studies have demonstrated the efficacy of copper surfaces in eradicating MDRO and *Clostridioides difficile* [34,35]. Since both MDRO and *Clostridioides difficile* infections represent only a fraction of global HAI, it may be so that the analyzed studies may not have been sufficiently powered to detect significant differences.

Similar, non-significant results on the effect of copper in reducing HAI have been reported by other studies. von Dessauer et al [36] in a non-RCT conducted in pediatric ICUs could not demonstrate a statistically significant reduction of HAI with the use of copper surfaces.

The results of our analysis should be interpreted keeping in mind several factors. To assess the incidence of HAI in two different cohorts, the importance of consistency in baseline factors cannot be underestimated. Except for two studies, all trials in our analysis were quasi-experimental studies. The different periods of intervention and control could not have guaranteed similar baseline characteristics of study patients in both groups. The difference in patient comorbidities, use of catheters, antibiotics, etc could have influenced outcomes. Such heterogeneity and complexity of studies evaluating the role of copper surfaces in reducing HAI has been reported by Chyderiotis et al [37] in their systematic review. On detailed analysis of forest plot of the primary outcome in our review, it is evident that the studies of Sifri et al [25] and Madden et al [23] were outliners. While Sifri et al [25] found significant benefit of copper-impregnated linen in reducing HAI, Madden et al [23] did not report any such difference. In the trial of Madden et al [23], the authors reported poorer hand hygiene compliance during the intervention period as compared to the control period (90.9% vs 95.3%). HAI is a complex process which is not only influenced by patient factors, but also basic infection-control methods practiced in the health-care setup. Leniency in basic measures may potentially nullify any possible effect of antimicrobial impregnated surfaces, limiting the ability of a study to draw strong evidence. This may be one of the reasons for lack of statistically significant results of Madden et al [23]. On the other hand, while all trials changed only basic hospital linen consisting of patient clothing, bedsheets, pillow covers, blankets, towels, etc to copper-impregnated linen in their studies, the same was not true for the trial of Sifri et al [25]. In the study of Sifri et al [25], not only linen but hard surfaces were also changed to copper-alloy surfaces. This may be a significant factor explaining the higher reduction of HAI (IRR:0.17, 95% CI:0.16–0.17) in their study. There were other inter-study variations seen in our review which included the difference in study setups, patient population, study duration and the total number of hospitalization days. These could have contributed to the large heterogeneity seen in our meta-analysis. However, in the meta-regression analysis, we could not find any influence of study duration and number of hospitalization days on the overall effect size.

The overall quality of studies was not high, which downgrades the evidence of our analysis. Blinding of hospital personnel was carried out in only two studies [22,26]. It is possible that in the absence of blinding, there may have been a degradation of infection control practices by health-care workers due to a sense of security offered by antimicrobial-impregnated linens. Similar negative correlations between glove use and hand hygiene compliance have been noted in the literature [38]. Also four of the included studies were longitudinal studies where regular hospital linen was replaced by copper linen. As the incidence of HAI can vary with changes in virulence of bacteria over the years, absence of control group for the same duration can be a significant limitation and this may have skewed the results of our review.

Despite, the above-mentioned limitations, our study is the first meta-analysis evaluating the role of copper-impregnated linen in reducing HAI. Only controlled studies were included in our analysis to avoid the possible bias of before-after studies. Also, the stability of our results on sensitivity analysis lends some support to our conclusions.

To conclude, we believe that current evidence on the use of copper-impregnated linen to reduce HAI is at best, conflicting. Our results indicate that copper-impregnated linen may reduce HAI, but there are still no evidences that copper-impregnated linens effectively reduced MDRO or *Clostridioides difficile* infections. The overall quality of evidence is also not high. Further, homogenous high-quality studies are required to strengthen the evidence on this subject.

## Supporting information

S1 Checklist Prisma checklist. (DOC)

S1 File Search strategy and results of PubMed database. (DOCX)

## References

[1] Magill SS, Edwards JR, Bamberg W, Beldavs ZG, Dumyati G, Kainer MA, et al Multistate point-prevalence survey of health care-associated infections. N Engl J Med. 2014;370: 1198–208. 10.1056/NEJMoa1306801 24670166 PMC4648343

[2] Wang J, Liu F, Tartari E, Huang J, Harbarth S, Pittet D, et al The Prevalence of Healthcare-Associated Infections in Mainland China: A Systematic Review and Meta-analysis. Infect Control Hosp Epidemiol. 2018;39: 701–709. 10.1017/ice.2018.60 29655388

[3] Marchetti A, Rossiter R. Economic burden of healthcare-associated infection in US acute care hospitals: societal perspective. J Med Econ. 2013;16: 1399–404. 10.3111/13696998.2013.842922 24024988

[4] Maragakis LL, Perencevich EN, Cosgrove SE. Clinical and economic burden of antimicrobial resistance. Expert Review of Anti-Infective Therapy. Expert Rev Anti Infect Ther; 2008;6: 751–763. 10.1586/14787210.6.5.751 18847410

[5] Oluwagbemiga AO, Akinsete SJ, Ana GR. Building conditions and the risk of nosocomial infection from microbial contamination of hospital appliances in a health care facility. Int J Environ Health Res. 2017;27: 264–275. 10.1080/09603123.2017.1332350 28553878

[6] Fijan S, Turk SŠ. Hospital textiles, are they a possible vehicle for healthcare-associated infections? Int J Environ Res Public Health. 2012;9: 3330–43. 10.3390/ijerph9093330 23202690 PMC3499872

[7] Ohl M, Schweizer M, Graham M, Heilmann K, Boyken L, Diekema D. Hospital privacy curtains are frequently and rapidly contaminated with potentially pathogenic bacteria. Am J Infect Control. 2012;40: 904–6. 10.1016/j.ajic.2011.12.017 22464039

[8] Mitchell A, Spencer M, Edmiston C. Role of healthcare apparel and other healthcare textiles in the transmission of pathogens: a review of the literature. J Hosp Infect. 2015;90: 285–92. 10.1016/j.jhin.2015.02.017 25935701 PMC7132459

[9] Arendsen LP, Thakar R, Sultan AH. The Use of Copper as an Antimicrobial Agent in Health Care, Including Obstetrics and Gynecology. Clin Microbiol Rev. 2019;32: e00125–18. 10.1128/CMR.00125-18 31413046 PMC6730497

[10] Zerbib S, Vallet L, Muggeo A, de Champs C, Lefebvre A, Jolly D, et al Copper for the Prevention of Outbreaks of Health Care-Associated Infections in a Long-term Care Facility for Older Adults. J Am Med Dir Assoc. 2020;21: 68-71.e1. 10.1016/j.jamda.2019.02.003 30954421

[11] Colin M, Klingelschmitt F, Charpentier E, Josse J, Kanagaratnam L, De Champs C, et al Copper alloy touch surfaces in healthcare facilities: An effective solution to prevent bacterial spreading. Materials (Basel). 2018;11: 2479 10.3390/ma11122479 30563265 PMC6317222

[12] Salgado CD, Sepkowitz KA, John JF, Cantey JR, Attaway HH, Freeman KD, et al Copper surfaces reduce the rate of healthcare-acquired infections in the intensive care unit. Infect Control Hosp Epidemiol. 2013;34: 479–86. 10.1086/670207 23571364

[13] Lazary A, Weinberg I, Vatine J-J, Jefidoff A, Bardenstein R, Borkow G, et al Reduction of healthcare-associated infections in a long-term care brain injury ward by replacing regular linens with biocidal copper oxide impregnated linens. Int J Infect Dis. 2014;24: 23–9. 10.1016/j.ijid.2014.01.022 24614137

[14] Pineda I, Hubbard R, Rodriguez F. The role of copper surfaces in reducing the incidence of healthcare-associated infections: A systematic review and meta-analysis. Can J Infect Control. 2017;32: 13–24. Available: http://www.greylit.org/

[15] Moher D, Liberati A, Tetzlaff J, Altman DG, PRISMA Group. Preferred Reporting Items for Systematic Reviews and Meta-Analyses: The PRISMA Statement. PLoS Med. 2009;6: e1000097 10.1371/journal.pmed.1000097 19621072 PMC2707599

[16] Higgins J, Thomas J, Chandler J, Cumpston M, Li T, Page M, et al Cochrane Handbook for Systematic Reviews of Interventions. Version 6. Cochrane Handbook for Systematic Reviews of Interventions. Cochrane; 2019 10.1002/9781119536604

[17] Kim SY, Park JE, Lee YJ, Seo H-J, Sheen S-S, Hahn S, et al Testing a tool for assessing the risk of bias for nonrandomized studies showed moderate reliability and promising validity. J Clin Epidemiol. 2013;66: 408–14. 10.1016/j.jclinepi.2012.09.016 23337781

[18] Suurmond R, van Rhee H, Hak T. Introduction, comparison, and validation of Meta-Essentials: A free and simple tool for meta-analysis. Research Synthesis Methods. Res Synth Methods; 2017;8: 537–553. 10.1002/jrsm.1260 28801932 PMC5725669

[19] Schweizer M, Graham M, Ohl M, Heilmann K, Boyken L, Diekema D. Novel hospital curtains with antimicrobial properties: a randomized, controlled trial. Infect Control Hosp Epidemiol. 2012;33: 1081–5. 10.1086/668022 23041804

[20] Irene G, Georgios P, Ioannis C, Anastasios T, Diamantis P, Marianthi C, et al Copper-coated textiles: armor against MDR nosocomial pathogens. Diagn Microbiol Infect Dis. 2016;85: 205–9. 10.1016/j.diagmicrobio.2016.02.015 27055400

[21] Burke GH, Butler JP. Analysis of the role of copper impregnated composite hard surfaces, bed linens and patient gowns in reducing healthcare-associated infection rates. Int J Infect Control. 2018;14: 1–8. 10.3396/IJIC.v14i1.005.18

[22] Marik PE, Shankaran S, King L. The effect of copper-oxide-treated soft and hard surfaces on the incidence of healthcare-associated infections: a two-phase study. J Hosp Infect. 2020;105: 265–271. 10.1016/j.jhin.2020.02.006 32068014

[23] Madden GR, Heon BE, Sifri CD. Effect of copper-impregnated linens on multidrug-resistant organism acquisition and Clostridium difficile infection at a long-term acute-care hospital. Infect Control Hosp Epidemiol. 2018;39: 1384–1386. 10.1017/ice.2018.196 30231949 PMC7063582

[24] JP B. Effect of Copper-Impregnated Composite Bed Linens and Patient Gowns on Healthcare-Associated Infection Rates in Six Hospitals. J Hosp Infect. 2018;100: e130–134 10.1016/j.jhin.2018.05.013 29803808

[25] Sifri CD, Burke GH, Enfield KB. Reduced health care-associated infections in an acute care community hospital using a combination of self-disinfecting copper-impregnated composite hard surfaces and linens. Am J Infect Control. 2016;44: 1565–1571. 10.1016/j.ajic.2016.07.007 27692785

[26] Marcus EL, Yosef H, Borkow G, Caine Y, Sasson A, Moses AE. Reduction of health care–associated infection indicators by copper oxide–impregnated textiles: Crossover, double-blind controlled study in chronic ventilator-dependent patients. Am J Infect Control. 2017;45: 401–403. 10.1016/j.ajic.2016.11.022 28034536

[27] Embry FC, Chinnes LF. Draft definitions for surveillance of infections in home health care. Am J Infect Control. 2000;28: 449–53. 10.1067/mic.2000.112150 11114614

[28] Stone ND, Ashraf MS, Calder J, Crnich CJ, Crossley K, Drinka PJ, et al Surveillance definitions of infections in long-term care facilities: revisiting the McGeer criteria. Infect Control Hosp Epidemiol. 2012;33: 965–77. 10.1086/667743 22961014 PMC3538836

[29] Schreiber PW, Sax H, Wolfensberger A, Clack L, Kuster SP, Swissnoso. The preventable proportion of healthcare-associated infections 2005–2016: Systematic review and meta-analysis. Infect Control Hosp Epidemiol. 2018;39: 1277–1295. 10.1017/ice.2018.183 30234463

[30] Magill SS, O’Leary E, Janelle SJ, Thompson DL, Dumyati G, Nadle J, et al Changes in Prevalence of Health Care-Associated Infections in U.S. Hospitals. N Engl J Med. 2018;379: 1732–1744. 10.1056/NEJMoa1801550 30380384 PMC7978499

[31] Borkow G, Gabbay J. Copper as a biocidal tool. Curr Med Chem. 2005;12: 2163–75. 10.2174/0929867054637617 16101497

[32] Montero DA, Arellano C, Pardo M, Vera R, Gálvez R, Cifuentes M, et al Antimicrobial properties of a novel copper-based composite coating with potential for use in healthcare facilities. Antimicrob Resist Infect Control. 2019;8: 3 10.1186/s13756-018-0456-4 30627427 PMC6321648

[33] MacLaurin A, Amaratunga K, Couris C, Frenette C, Galioto R, Hansen G, et al Measuring and Monitoring Healthcare-Associated Infections: A Canadian Collaboration to Better Understand the Magnitude of the Problem. Healthc Q. 2020;22: 116–128. 10.12927/hcq.2020.26040 32049622

[34] Weaver L, Michels HT, Keevil CW. Survival of Clostridium difficile on copper and steel: futuristic options for hospital hygiene. J Hosp Infect. 2008;68: 145–151. 10.1016/j.jhin.2007.11.011 18207284

[35] Noyce JO, Michels H, Keevil CW. Potential use of copper surfaces to reduce survival of epidemic meticillin-resistant Staphylococcus aureus in the healthcare environment. J Hosp Infect. 2006;63: 289–297. 10.1016/j.jhin.2005.12.008 16650507

[36] von Dessauer B, Navarrete MS, Benadof D, Benavente C, Schmidt MG. Potential effectiveness of copper surfaces in reducing health care-associated infection rates in a pediatric intensive and intermediate care unit: A nonrandomized controlled trial. Am J Infect Control. 2016;44: e133–9. 10.1016/j.ajic.2016.03.053 27318524

[37] Chyderiotis S, Legeay C, Verjat-Trannoy D, Le Gallou F, Astagneau P, Lepelletier D. New insights on antimicrobial efficacy of copper surfaces in the healthcare environment: a systematic review. Clinical Microbiology and Infection. Clin Microbiol Infect; 2018;24: 1130–1138. 10.1016/j.cmi.2018.03.034 29605564

[38] Cusini A, Nydegger D, Kaspar T, Schweiger A, Kuhn R, Marschall J. Improved hand hygiene compliance after eliminating mandatory glove use from contact precautions-Is less more? Am J Infect Control. 2015;43: 922–7. 10.1016/j.ajic.2015.05.019 26122873

