# Supplementary material for: Efficacy of copper-impregnated hospital linen in reducing healthcare-associated infections: A systematic review and meta-analysis
Source: PLoS One. 2020 Jul 20;15(7):e0236184. doi: 10.1371/journal.pone.0236184 (PMC7371175; doi:10.1371/journal.pone.0236184)
Supplement: S1 File — (DOCX) [file pone.0236184.s002.docx]

Supplementary material 1: Search strategy and results of PubMed database

| **Search number** | **Query** | **Search Details** | **Results** |
| --- | --- | --- | --- |
| **1** | ((copper) AND (clothing)) AND (infection) | (((("copper"[MeSH Terms] OR "copper"[All Fields]) OR "coppers"[All Fields]) OR "copper s"[All Fields]) AND (((((((("clothing"[MeSH Terms] OR "clothing"[All Fields]) OR "clothes"[All Fields]) OR "clothings"[All Fields]) OR "textiles"[MeSH Terms]) OR "textiles"[All Fields]) OR "cloth"[All Fields]) OR "clothed"[All Fields]) OR "cloths"[All Fields])) AND ((((((((((((((((((((("infect"[All Fields] OR "infectability"[All Fields]) OR "infectable"[All Fields]) OR "infectant"[All Fields]) OR "infectants"[All Fields]) OR "infected"[All Fields]) OR "infecteds"[All Fields]) OR "infectibility"[All Fields]) OR "infectible"[All Fields]) OR "infecting"[All Fields]) OR "infection s"[All Fields]) OR "infections"[MeSH Terms]) OR "infections"[All Fields]) OR "infection"[All Fields]) OR "infective"[All Fields]) OR "infectiveness"[All Fields]) OR "infectives"[All Fields]) OR "infectivities"[All Fields]) OR "infects"[All Fields]) OR "pathogenicity"[MeSH Subheading]) OR "pathogenicity"[All Fields]) OR "infectivity"[All Fields]) | 41 |
| **2** | ((copper) AND (linen)) AND (infection) | (((("copper"[MeSH Terms] OR "copper"[All Fields]) OR "coppers"[All Fields]) OR "copper s"[All Fields]) AND (((("bedding and linens"[MeSH Terms] OR ("bedding"[All Fields] AND "linens"[All Fields])) OR "bedding and linens"[All Fields]) OR "linen"[All Fields]) OR "linens"[All Fields])) AND ((((((((((((((((((((("infect"[All Fields] OR "infectability"[All Fields]) OR "infectable"[All Fields]) OR "infectant"[All Fields]) OR "infectants"[All Fields]) OR "infected"[All Fields]) OR "infecteds"[All Fields]) OR "infectibility"[All Fields]) OR "infectible"[All Fields]) OR "infecting"[All Fields]) OR "infection s"[All Fields]) OR "infections"[MeSH Terms]) OR "infections"[All Fields]) OR "infection"[All Fields]) OR "infective"[All Fields]) OR "infectiveness"[All Fields]) OR "infectives"[All Fields]) OR "infectivities"[All Fields]) OR "infects"[All Fields]) OR "pathogenicity"[MeSH Subheading]) OR "pathogenicity"[All Fields]) OR "infectivity"[All Fields]) | 11 |
| **3** | ((copper) AND (bedsheets)) AND (infection) | ("copper"[All Fields] AND "bedsheets"[All Fields]) AND "infection"[All Fields] | 0 |
| **4** | ((copper) AND (dress)) AND (infection) | (((("copper"[MeSH Terms] OR "copper"[All Fields]) OR "coppers"[All Fields]) OR "copper s"[All Fields]) AND (((((((((("bandages"[MeSH Terms] OR "bandages"[All Fields]) OR "dressing"[All Fields]) OR "dressings"[All Fields]) OR "dressed"[All Fields]) OR "dresses"[All Fields]) OR "dressing s"[All Fields]) OR "drug hypersensitivity syndrome"[MeSH Terms]) OR (("drug"[All Fields] AND "hypersensitivity"[All Fields]) AND "syndrome"[All Fields])) OR "drug hypersensitivity syndrome"[All Fields]) OR "dress"[All Fields])) AND ((((((((((((((((((((("infect"[All Fields] OR "infectability"[All Fields]) OR "infectable"[All Fields]) OR "infectant"[All Fields]) OR "infectants"[All Fields]) OR "infected"[All Fields]) OR "infecteds"[All Fields]) OR "infectibility"[All Fields]) OR "infectible"[All Fields]) OR "infecting"[All Fields]) OR "infection s"[All Fields]) OR "infections"[MeSH Terms]) OR "infections"[All Fields]) OR "infection"[All Fields]) OR "infective"[All Fields]) OR "infectiveness"[All Fields]) OR "infectives"[All Fields]) OR "infectivities"[All Fields]) OR "infects"[All Fields]) OR "pathogenicity"[MeSH Subheading]) OR "pathogenicity"[All Fields]) OR "infectivity"[All Fields]) | 31 |
| 5 | ((copper) AND (clothing)) AND (antimicrobial) | (((("copper"[MeSH Terms] OR "copper"[All Fields]) OR "coppers"[All Fields]) OR "copper s"[All Fields]) AND (((((((("clothing"[MeSH Terms] OR "clothing"[All Fields]) OR "clothes"[All Fields]) OR "clothings"[All Fields]) OR "textiles"[MeSH Terms]) OR "textiles"[All Fields]) OR "cloth"[All Fields]) OR "clothed"[All Fields]) OR "cloths"[All Fields])) AND (((((("anti infective agents"[Pharmacological Action] OR "anti-infective agents"[MeSH Terms]) OR ("anti infective"[All Fields] AND "agents"[All Fields])) OR "anti infective agents"[All Fields]) OR "antimicrobial"[All Fields]) OR "antimicrobials"[All Fields]) OR "antimicrobially"[All Fields]) | 82 |
| 6 | ((copper) AND (linen)) AND (antimicrobial) | (((("copper"[MeSH Terms] OR "copper"[All Fields]) OR "coppers"[All Fields]) OR "copper s"[All Fields]) AND (((("bedding and linens"[MeSH Terms] OR ("bedding"[All Fields] AND "linens"[All Fields])) OR "bedding and linens"[All Fields]) OR "linen"[All Fields]) OR "linens"[All Fields])) AND (((((("anti infective agents"[Pharmacological Action] OR "anti-infective agents"[MeSH Terms]) OR ("anti infective"[All Fields] AND "agents"[All Fields])) OR "anti infective agents"[All Fields]) OR "antimicrobial"[All Fields]) OR "antimicrobials"[All Fields]) OR "antimicrobially"[All Fields]) | 6 |
| 7 | ((copper) AND (bedsheets)) AND (antimicrobial) | ("copper"[All Fields] AND "bedsheets"[All Fields]) AND "antimicrobial"[All Fields] | 0 |
| 8 | ((copper) AND (dress)) AND (antimicrobial) | (((("copper"[MeSH Terms] OR "copper"[All Fields]) OR "coppers"[All Fields]) OR "copper s"[All Fields]) AND (((((((((("bandages"[MeSH Terms] OR "bandages"[All Fields]) OR "dressing"[All Fields]) OR "dressings"[All Fields]) OR "dressed"[All Fields]) OR "dresses"[All Fields]) OR "dressing s"[All Fields]) OR "drug hypersensitivity syndrome"[MeSH Terms]) OR (("drug"[All Fields] AND "hypersensitivity"[All Fields]) AND "syndrome"[All Fields])) OR "drug hypersensitivity syndrome"[All Fields]) OR "dress"[All Fields])) AND (((((("anti infective agents"[Pharmacological Action] OR "anti-infective agents"[MeSH Terms]) OR ("anti infective"[All Fields] AND "agents"[All Fields])) OR "anti infective agents"[All Fields]) OR "antimicrobial"[All Fields]) OR "antimicrobials"[All Fields]) OR "antimicrobially"[All Fields]) | 49 |
| 9 | ((copper) AND (hospital)) AND (infection) | (((("copper"[MeSH Terms] OR "copper"[All Fields]) OR "coppers"[All Fields]) OR "copper s"[All Fields]) AND (((((((((((((("hospital s"[All Fields] OR "hospitalisation"[All Fields]) OR "hospitalization"[MeSH Terms]) OR "hospitalization"[All Fields]) OR "hospitalised"[All Fields]) OR "hospitalising"[All Fields]) OR "hospitality"[All Fields]) OR "hospitalisations"[All Fields]) OR "hospitalizations"[All Fields]) OR "hospitalize"[All Fields]) OR "hospitalized"[All Fields]) OR "hospitalizing"[All Fields]) OR "hospitals"[MeSH Terms]) OR "hospitals"[All Fields]) OR "hospital"[All Fields])) AND ((((((((((((((((((((("infect"[All Fields] OR "infectability"[All Fields]) OR "infectable"[All Fields]) OR "infectant"[All Fields]) OR "infectants"[All Fields]) OR "infected"[All Fields]) OR "infecteds"[All Fields]) OR "infectibility"[All Fields]) OR "infectible"[All Fields]) OR "infecting"[All Fields]) OR "infection s"[All Fields]) OR "infections"[MeSH Terms]) OR "infections"[All Fields]) OR "infection"[All Fields]) OR "infective"[All Fields]) OR "infectiveness"[All Fields]) OR "infectives"[All Fields]) OR "infectivities"[All Fields]) OR "infects"[All Fields]) OR "pathogenicity"[MeSH Subheading]) OR "pathogenicity"[All Fields]) OR "infectivity"[All Fields]) | 664 |
| **10** | (antimicrobial) AND (clothing) | (((((("anti infective agents"[Pharmacological Action] OR "anti-infective agents"[MeSH Terms]) OR ("anti infective"[All Fields] AND "agents"[All Fields])) OR "anti infective agents"[All Fields]) OR "antimicrobial"[All Fields]) OR "antimicrobials"[All Fields]) OR "antimicrobially"[All Fields]) AND (((((((("clothing"[MeSH Terms] OR "clothing"[All Fields]) OR "clothes"[All Fields]) OR "clothings"[All Fields]) OR "textiles"[MeSH Terms]) OR "textiles"[All Fields]) OR "cloth"[All Fields]) OR "clothed"[All Fields]) OR "cloths"[All Fields]) | 2,617 |
| **11** | (antimicrobial) and (linen) | (((((("anti infective agents"[Pharmacological Action] OR "anti-infective agents"[MeSH Terms]) OR ("anti infective"[All Fields] AND "agents"[All Fields])) OR "anti infective agents"[All Fields]) OR "antimicrobial"[All Fields]) OR "antimicrobials"[All Fields]) OR "antimicrobially"[All Fields]) AND (((("bedding and linens"[MeSH Terms] OR ("bedding"[All Fields] AND "linens"[All Fields])) OR "bedding and linens"[All Fields]) OR "linen"[All Fields]) OR "linens"[All Fields]) | 398 |
| **12** | (antimicrobial) AND (bedsheets) | (((((("anti infective agents"[Pharmacological Action] OR "anti-infective agents"[MeSH Terms]) OR ("anti infective"[All Fields] AND "agents"[All Fields])) OR "anti infective agents"[All Fields]) OR "antimicrobial"[All Fields]) OR "antimicrobials"[All Fields]) OR "antimicrobially"[All Fields]) AND ("bedsheet"[All Fields] OR "bedsheets"[All Fields]) | 1 |
| **13** | ((antimicrobial) AND (hospital)) AND (dress) | ((((((("anti infective agents"[Pharmacological Action] OR "anti-infective agents"[MeSH Terms]) OR ("anti infective"[All Fields] AND "agents"[All Fields])) OR "anti infective agents"[All Fields]) OR "antimicrobial"[All Fields]) OR "antimicrobials"[All Fields]) OR "antimicrobially"[All Fields]) AND (((((((((((((("hospital s"[All Fields] OR "hospitalisation"[All Fields]) OR "hospitalization"[MeSH Terms]) OR "hospitalization"[All Fields]) OR "hospitalised"[All Fields]) OR "hospitalising"[All Fields]) OR "hospitality"[All Fields]) OR "hospitalisations"[All Fields]) OR "hospitalizations"[All Fields]) OR "hospitalize"[All Fields]) OR "hospitalized"[All Fields]) OR "hospitalizing"[All Fields]) OR "hospitals"[MeSH Terms]) OR "hospitals"[All Fields]) OR "hospital"[All Fields])) AND (((((((((("bandages"[MeSH Terms] OR "bandages"[All Fields]) OR "dressing"[All Fields]) OR "dressings"[All Fields]) OR "dressed"[All Fields]) OR "dresses"[All Fields]) OR "dressing s"[All Fields]) OR "drug hypersensitivity syndrome"[MeSH Terms]) OR (("drug"[All Fields] AND "hypersensitivity"[All Fields]) AND "syndrome"[All Fields])) OR "drug hypersensitivity syndrome"[All Fields]) OR "dress"[All Fields]) | 1,857 |
